# Microbial Community Dynamics in Early Tufa Biofilms

**DOI:** 10.1002/mbo3.70153

**Published:** 2025-11-17

**Authors:** Andrea Čačković, Andrijana Brozinčević, Marija Mirosavljević, Sandi Orlić

**Affiliations:** ^1^ Division of Materials Chemistry Ruđer Bošković Institute Zagreb Croatia; ^2^ Scientific Research Center “Dr. Ivo Pevalek,” Plitvice Lakes National Park Plitvička Jezera Croatia; ^3^ Center of Excellence for Science and Technology‐Integration of Mediterranean Region (STIM) Zagreb Croatia; ^4^ University of Montenegro Podgorica Montenegro

**Keywords:** biofilm, carbonate precipitation, functional annotation, microeukaryotic communities, prokaryotic communities, tufa barriers

## Abstract

Karst freshwater systems represent unique ecological niches where physicochemical and biological interactions promote intensive calcium carbonate precipitation and the formation of tufa barriers. Here, we studied the composition, diversity, and functional potential of prokaryotic and microeukaryotic communities involved in early‐stage tufa formation at two sites within the Plitvice Lakes National Park, Croatia. Over a 5‐day period in two different seasons, tufa and water samples were collected at Prošćansko Lake and Novakovića Brod to examine temporal and spatial microbial dynamics. High‐throughput sequencing revealed early stabilization of prokaryotic communities within tufa biofilms, dominated by genera, such as *Bacillus*, *Delftia*, *Hyphomicrobium*, and *Methylobacterium–Methylorubrum*, which are linked to carbonate precipitation processes. With biofilm maturation, shifts toward *Acinetobacter* and *Rhodoferax* indicated increasing heterotrophic activity and organic matter degradation. In contrast, microeukaryotic communities were more variable, with diatoms and Zygnemophyceae contributing to extracellular polymeric substance production, crucial for carbonate entrapment. Site‐specific patterns reflected environmental influences, such as hydrodynamics and terrestrial organic input. The results underscore the importance of microbial succession and community specialization in the formation and stability of tufa barriers, offering new insights into microbial contributions to biogeochemical processes in karst freshwater systems.

## Graphical abstract

Microbial succession shapes the early development of tufa biofilms in karst freshwater systems, with prokaryotic communities stabilizing rapidly and microeukaryotes contributing to extracellular polymeric substance production and carbonate entrapment. Site‐specific hydrodynamics and organic inputs drive community specialization, ultimately influencing tufa barrier formation and stability.

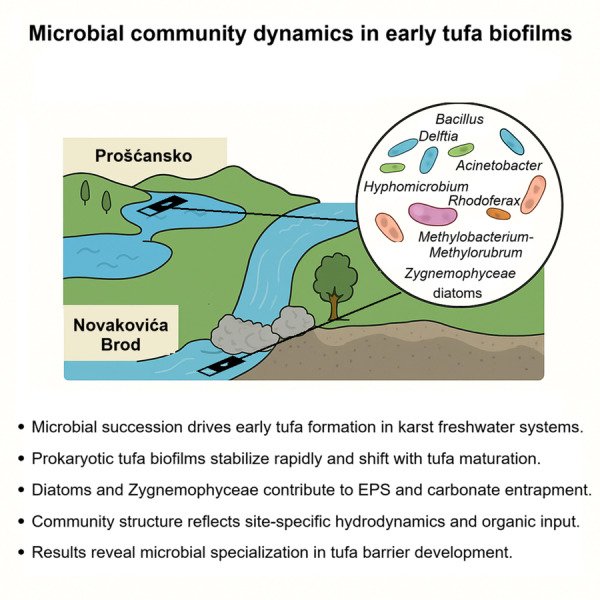

## 1 Introduction

Karst streams are dynamic lotic habitats and biodiversity hotspots shaped by interactions between hydrology, geology, and biology (Simović et al. 2024). Karst streams lie on the bedrock of carbonate rocks (De Waele and Gutiérrez 2022), where high CO_2_ concentrations in groundwater lead to calcium carbonate (CaCO_3_) supersaturation. When water reaches the surface, CO_2_ spontaneously degasses, particularly at more turbulent locations, such as waterfalls (Kis et al. 2020), causing CaCO_3_ precipitation and formation of tufa. Tufa is a deposit of CaCO_3_ precipitation occurring at the abovementioned and other specific physicochemical conditions, such as ambient water temperature, alkaline pH (> 8.0), dissolved organic matter (DOM) concentrations below 10 mg/L C, and flow rate of 2–80 L/s (Capezzuoli et al. 2014).

Tufa deposits consist of CaCO_3_ encrusting organic material, primarily produced by various periphyton microorganisms, such as cyanobacteria, algae, bacteria, protozoa, and multicellular microorganisms (Gulin et al. 2021). Due to their short life cycles, these communities can change significantly in a very short time, sometimes even within 24 h (Wu 2016). Microbes play multiple roles (Shiraishi et al. 2008), including producing extracellular polymeric substance (EPS) for trapping suspended particles from the surrounding water, initiating the encrustation process (Sun et al. 2020), facilitating mineralization (Li et al. 2023), and, through photosynthesis, promoting calcite precipitation around cell surfaces (Matoničkin Kepčija and Miliša 2023). While cyanobacteria and diatoms are well established as major contributors to tufa formation (Schneider et al. 2015; Winsborough 2000), molecular studies have revealed a broader microbial diversity, including heterotrophic prokaryotes and microeukaryotes involved in DOM degradation and EPS decomposition (Manzo et al. 2012). Moreover, their composition is dynamic and can shift with changes in water flow (Li et al. 2023).

Despite growing knowledge, most studies have focused on abundant taxa or long‐term community composition. Little is known about the short‐term dynamics of prokaryotic and microeukaryotic communities during the initial stages of tufa biofilm development, when microbial activity and EPS production drive the earliest steps of mineral entrapment.

In this study, we analyzed the composition of prokaryote and microeukaryote communities within the tufa biofilms of the Plitvice Lakes. This freshwater network ecosystem, consisting of rivers, streams, and lakes interconnected by tufa barriers that form waterfalls, provides a unique habitat. Two locations were selected within the system, at the exit and entrance points of the lakes, where sedimentation occurs. Tufa samples were collected over a 5‐day period, alongside water samples from the surrounding environment, to provide a comprehensive two‐seasonal overview of these communities.

## 2 Materials and Methods

### 2.1 Study Area and Sampling

Plitvice Lakes, located in the Dinaric karst region of Croatia, form a 9.5‐km aquatic system of 16 cascading lakes separated by tufa barriers. The upper 12 lakes lie in dolomite, while the lower four flow through a limestone canyon (Bočić et al. 2023). The entire lake system is supplied with water by the Bijela and Crna rivers, which merge into the Matica River and enter Lake Prošćansko, continuing through Lake Kozjak to Lake Novakovića Brod, before draining into the Korana River, along with water from the Plitvice waterfall.

**Figure 1:**
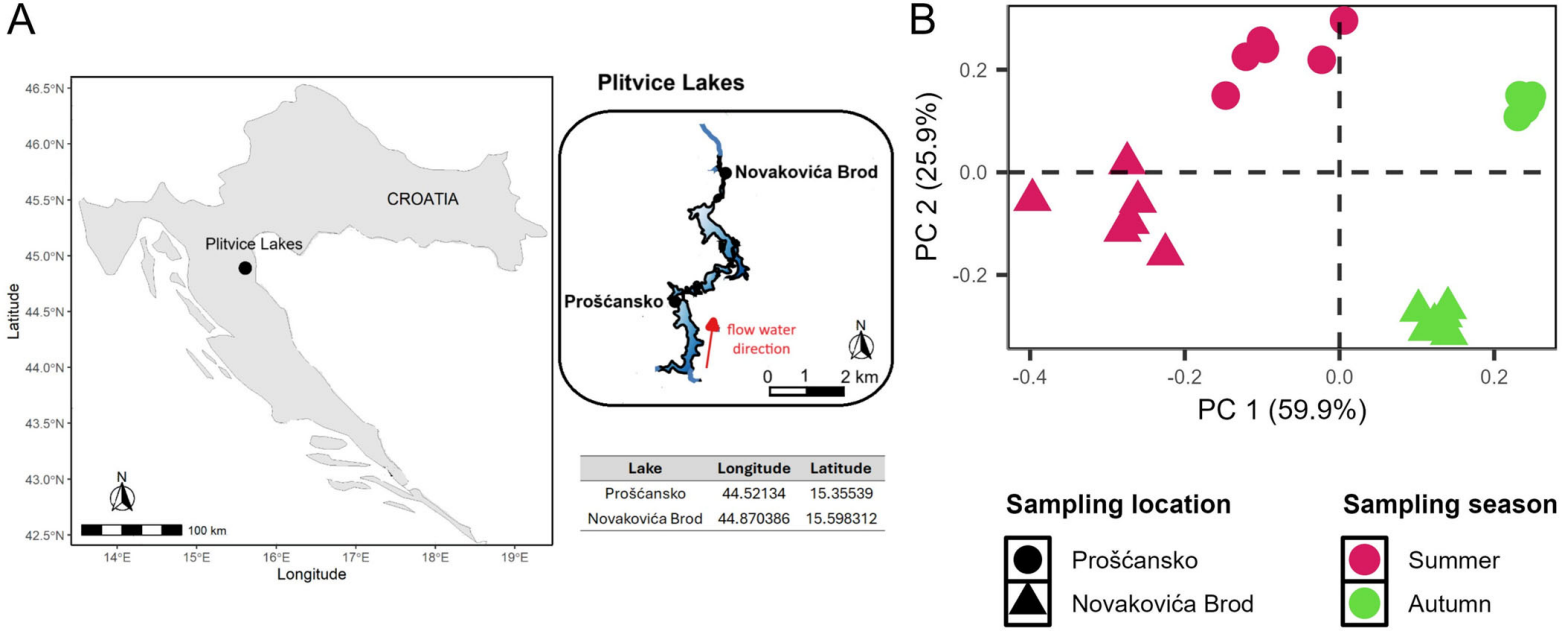
Study area. (A) Geographical position of sampled locations in Plitvice lakes, located in Croatia. (B) Principal component analysis ordination on the environmental variables during the investigated period. Sampling seasons are color‐coded and sampling locations are shaped marked. PC, polycarbonate.

Two locations (Figure 1A) were selected as research sites: at the outflow of Lake Prošćansko (44.52134, 15.35539), on a moss‐covered waterfall, and at the inflow to Lake Novakovića Brod (44.870386, 15.598312), on low cascades with less moss. Holders with microscopic slides were placed at 1 m depth and collected after 12, 24, 48, 72, 96, and 120 h in two seasons. At each time point, one slide was frozen (–20°C) and another was fixed with 1% formaldehyde (1 h, room temperature) before freezing.

During sampling, a multisensor probe was used to measure dissolved oxygen (DO), temperature, pH, and electrical conductivity (EC) in situ. At the same locations, 2 L of water samples were collected in polycarbonate (PC) bottles.

### 2.2 DNA Extraction, Amplification, and Sequencing

Every microscopic slide was placed in a 0.9% sodium chloride solution in a Falcon tube, and the biofilm material was scratch into solution, then filtered onto a 0.22‐mm‐pore‐size PC filter (Whatman Nuclepore Track‐Etch membrane; diameter, 47 mm) using a vacuum pump. Water samples were filtered directly onto a 0.22‐mm‐pore‐size PC filter. Total genomic DNA from filters was extracted with the DNeasy PowerSoil kit (Qiagen Inc., Valencia, CA, USA) following the manufacturer's protocol.

The hypervariable V4 region of the prokaryotic 16S ribosomal RNA (rRNA) gene was amplified by PCR using primer pair 515F Parada (5′‐GTGYCAGCMGCCGCGGTAA‐3′) (Parada et al. 2016) and 806R Apprill (5′‐GGACTACNVGGGTWTCTAAT‐3′) (Apprill et al. 2015). The hypervariable V9 region of the gene encoding 18S rRNA was amplified by PCR using primer pair for eukaryotic identification TAReuk454FWD1 (5′‐CCAGCASCYGCGGTAATTCC‐3′) (STOECK et al. 2010) and TAReukREV3mod (5′‐ACTTTCGTTCTTGATYRATGA‐3′) (Piredda et al. 2017). All samples were amplified, barcoded, purified, and prepared following the protocol (Pjevac et al. 2021) and sequenced on an Illumina MiSeq (v3 chemistry, 2 × 300 bp) at the Joint Microbiome Facility, Medical University of Vienna.

### 2.3 Data Processing

Individual amplicon pools were extracted from the raw sequencing data using the FASTQ workflow in BaseSpace (Illumina) with default settings, allowing one mismatch in 6‐nucleotide (nt) library indexes. PhiX contamination was removed from the input data using BBDuk (BBTools) (Bushnell 2014). Demultiplexing was verified using the Python package Demultiplex (Laros JFJ, github.com/jfjlaros/demultiplex) allowing up to two mismatches in the primers/linkers and one in the barcodes, subsequently trimmed with BBDuk. For 16S rRNA libraries, 47/48 bases were trimmed from F.1/R.2 and F.2/R.1; for 18S rRNA, 48/49 bases.

Amplicon Sequence Variants (ASVs) were inferred in R with DADA2 v1.14.1 (Callahan et al. 2016) in pooled mode using all amplicon libraries per sequencing run. Taxonomic assignment of 16S rRNA region V4 ASVs was based on the SILVA database SSU Ref NR 99, release 138.1 (https://www.ncbi.nlm.nih.gov/pubmed/23193283). For 18S rRNA region V9 ASVs, taxonomic classification was based on the PR2 reference database (v.4.12.0). Both assignments were conducted using SINA version 1.6.1 (https://www.ncbi.nlm.nih.gov/pubmed/22556368).

The sequencing of V4 16S rRNA resulted in 296,601 reads, and after filtering, the remaining 232,740 reads were clustered into 825 prokaryotic ASVs. The V9 18S rRNA sequencing resulted in 348,268 reads, with 313,158 retained after filtering, resulting in 765 eukaryotic ASVs.

### 2.4 Statistical Analysis

Resulted ASVs were statistically analyzed in R environment (version 4.3.3.) (R Core Team 2021). In the analysis, packages used were phyloseq (McMurdie and Holmes 2013), vegan (Oksanen et al. 2019), dplyr (Wickham et al. 2014), and ggplot2 (Wickham 2016).

The beta diversity of environmental parameters was calculated by performing a principal component analysis (PCA) on a distance matrix of *Z*‐score‐normalized data using vegan.

Before statistical analysis, ASVs classified as eukaryotes, mitochondria, or chloroplasts, unassigned ASVs at the phylum level, singletons, and doubletons in the 16S rRNA data set were removed, as well as unassigned ASVs at the supergroup level in the 18S rRNA data set.

From the 16S rRNA data set, four samples under 1000 reads were removed (water: Novakovića Brod summer 24 h, Prošćansko autumn 120 h, and Novakovića Brod autumn 96 h; tufa: Prošćansko autumn 12 h), leaving 42 samples for statistical analysis. From the 18S rRNA data set, nine samples under 100 reads were removed (water: Novakovića Brod summer 72 h, autumn 12 and 24 h; tufa: Prošćansko summer 48 h, autumn 12, 24, 72, 96, and 120 h, and Novakovića Brod autumn 24 h), resulting in 38 samples for statistical analysis.

Alpha diversity was computed by rarefaction on the data sets by subsampling libraries to the smallest library size and estimated as diversity according to the Shannon index (Shannon 1948) and richness.

Beta diversity was done using Bray–Curtis dissimilarity matrices and visualized via principal coordinate analysis. Significant community differences by sample type, location, season, and time were tested with PERMANOVA. The taxonomic abundance of the initial community was studied at the phylum level, including all taxa with relative abundance greater than 1%, while all the taxa with relative abundance less than 1% formed the “others” group. The taxonomic abundance of the Abundant taxa was studied at the genus/family level (> 10%).

Shared and unique ASVs of communities were depicted in a Venn diagram using the package ggVennDiagram (Gao et al. 2021).

Distance decay relationship visualization was based on Bray–Curtis similarity index after normalization of the data set through cumulative sum scaling with the metagenomeSeq package (Paulson et al. 2013). Geographic distance was measured using a “Vincenty” (ellipsoid) great circle method in packages enmSdm (Morelli et al. 2020) and geosphere (v1.5.10) (Hijmans 2010).

Community stability was calculated using the ASV table (Yuan et al. 2021) for every sampling hour within different seasons. Trends in prokaryotic and microeukaryotic stability were visualized using a generalized additive model for fitting (Wood 2011).

Functional prediction of prokaryotic communities was performed in the package “microeco” (Liu et al. 2021) using the Functional Annotation of Prokaryotic Taxa (FAPROTAX) database and grouped into broader ecological categories.

### 2.5 Scanning Electron Microscopy (SEM)

Formaldehyde‐fixed slides were dried at 60°C for 2 days, Au‐coated, and mounted with carbon tape. SAM imaging was performed at an Axia ChemiSEM electron microscope (Thermo Fisher Scientific Inc., Waltham, MA, USA) under high‐pressure mode at 5.0E−2 Pa, 5 and 10 kV accelerating voltage, and working distance of 10 mm. The Axia ChemiSEM SEM/EDS system and xT Microscope Control Software version v.27.4.0. were used for high‐resolution mapping and elemental analysis.

## 3 Results

### 3.1 Environmental Parameters of Water

Environmental parameters were measured at two locations, the exit of Lake Prošćansko and the entrance of Lake Novakovića Brod (Figure 1A), during summer and autumn. Rainfall was slightly higher in autumn (148.9 mm) than in summer (115.1 mm; Supporting Information Figure A1). Water temperature averaged 22°C in summer and 15.5°C in autumn at Novakovića Brod, while at Prošćansko it was 13°C in autumn and decreased from 21°C to 17°C during summer. pH values were generally stable (8.1–8.4). DO concentrations were higher in autumn at both locations (9.6–9.8 mg/L), although summer values at Prošćansko occasionally exceeded autumn levels. EC was consistently higher at Prošćansko, with both sites showing elevated values in summer. Water flow speed was also greater in summer, especially at Novakovića Brod, which also received the highest light intensity during this season.

PCA showed the difference among samples between sampling locations and seasons (Figure 1B). Additionally, summer samples from both locations were more dispersed, whereas autumn samples clustered closely depending on location.

### 3.2 Alpha Diversity of Microbial Communities

**Figure 2:**
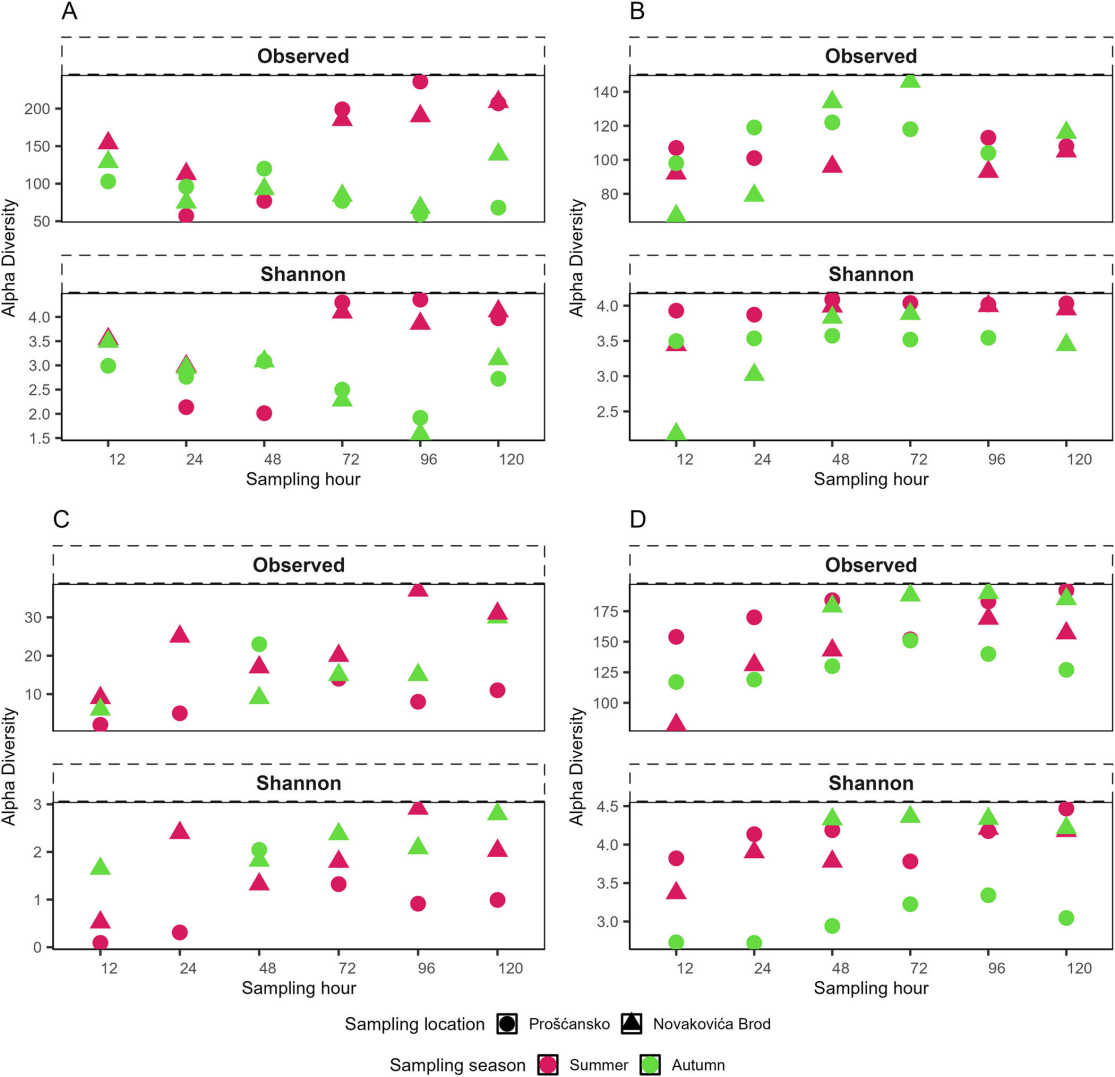
Alpha diversity of microbial communities. Alpha diversity indices of prokaryotic communities in tufa (A) and water (B) and microeukaryotic in tufa (C) and water (D) determined as Richness and Shannon index. Sampling seasons are color‐coded, and sampling locations are shaped marked.

Prokaryotic richness and diversity of tufa communities were generally higher in summer, except at midsampling (48 h) when autumn values were higher. Location‐specific patterns were observed, with Prošćansko showing elevated richness and diversity at several time points, while Novakovića Brod displayed overall higher values (Figure 2A). In water samples, richness was usually higher in autumn, especially at Prošćansko, whereas alpha diversity was greater in summer and at Prošćansko, except in autumn when Novakovića Brod showed higher values (Figure 2B).

Microeukaryotic richness in tufa was higher in summer at Novakovića Brod, while alpha diversity peaked in autumn, also at Novakovića Brod (Figure 2C). In water samples, richness was similar between seasons but shifted by location, with higher values at Prošćansko in summer and at Novakovića Brod in autumn. Alpha diversity reached its maximum in autumn at Novakovića Brod and was lowest at Prošćansko (Figure 2D).

### 3.3 Initial Microbial Communities

Multivariate analysis revealed that prokaryotic communities differed by sample type after 12 h (PERMANOVA: *R* ^2^ = 0.43, *p* = 0.027). Water communities differed seasonally and by location, while tufa communities were more consistent across sites and seasons (Figure 2A). Tufa communities were dominated by Proteobacteria, with Dependitae and Firmicutes. Water communities in summer were equally dominated by Actinobactetiota, Proteobacteria with Bacteroidota at Prošćansko lake and Cyanobacteria and Deinococcota at Novakovića Brod site. In autumn, water communities shifted to Firmicutes at Novakovića Brod and to Actinobacteriota, Cyanobacteria, Proteobacteria, and Verrucomicrobiota at Prošćansko (Figure 2B).

**Figure 3:**
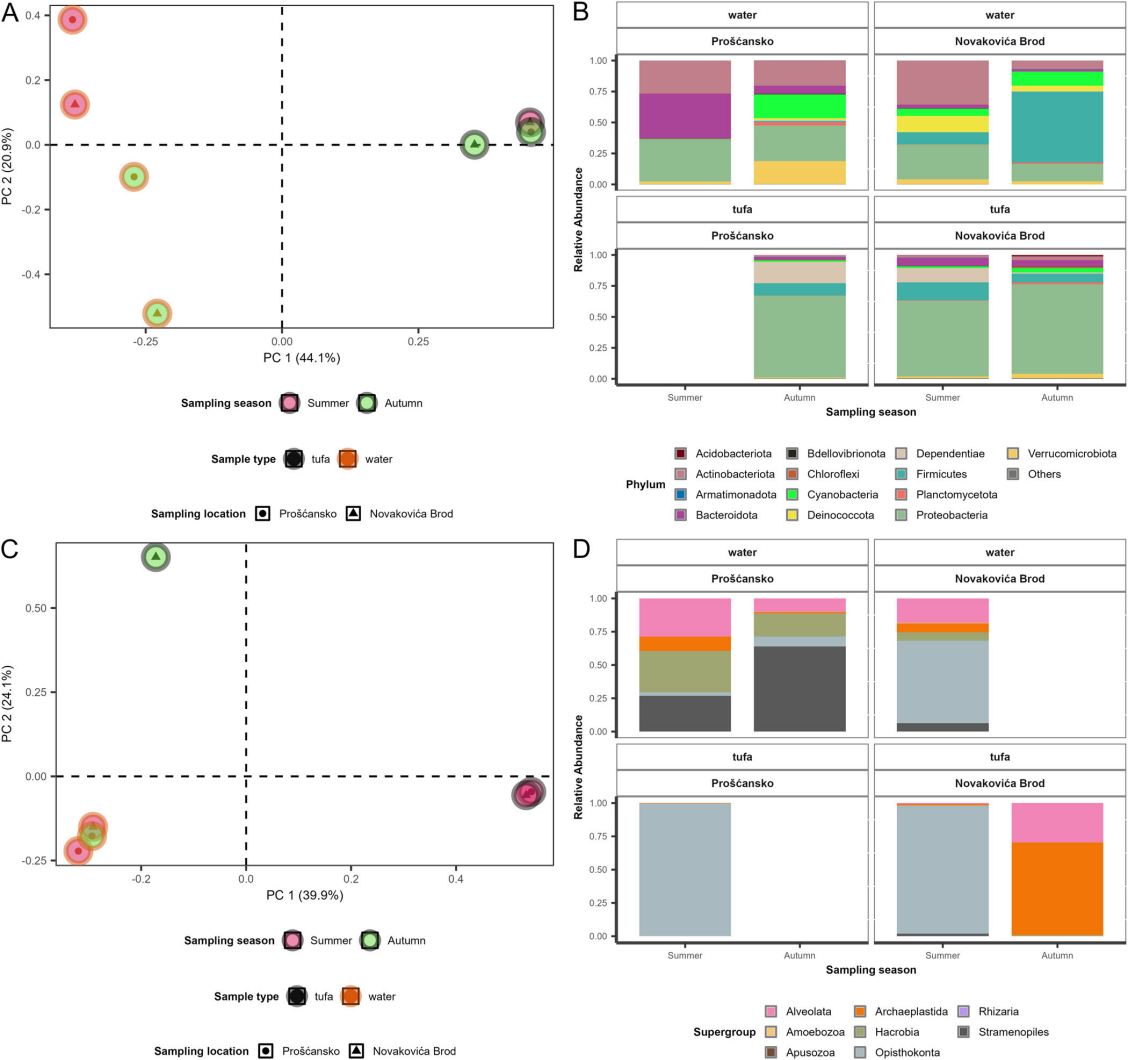
Significant difference in initial prokaryotic communities between water and tufa. (A) Principal coordinate analysis (PCoA) ordination showing Bray–Curtis dissimilarity in initial prokaryotic community compositions across different sample types, color coded by sampling season and shape coded by sampling location (see legend). (B) Average relative abundance of the initial prokaryotic community (phylum level) in different sample types, across two sampling seasons and locations. (C) PCoA ordination showing Bray–Curtis dissimilarity in initial microeukaryotic community compositions across different sample types, color coded by sampling season and shape coded by sampling location (see legend). (D) Average relative abundance of the initial microeukaryotic community (supergroup level) in different sample types, across two sampling seasons and locations. PC, polycarbonate.

Microeukaryotic communities showed no significant sample‐type differences at 12 h (PERMANOVA: *R* ^2^ = 0.34, *p* = 0.1). Tufa communities shifted seasonally, dominated by Opisthokonta in summer and Alveolata with Archaeplastida in autumn. Water communities consisted of Alveolata, Archaeplastida, Opisthokonta, and Stramenopiles, with seasonal and site‐specific variation (Figure 3D).

### 3.4 Tufa and Water Microbial Communities

Multivariate analysis showed that prokaryotic communities differed primarily by sample type (PERMANOVA: *R* ^2^ = 0.36, *p* = 0.001; Supporting Information Figure A2A), with stronger seasonal variation in water (*R* ^2^ = 0.46, *p* = 0.001; Supporting Information Figure A3A) than in tufa (*R* ^2^ = 0.09, *p* = 0.036; Supporting Information Figure A3B). Microeukaryotic communities were also separated by sample type (PERMANOVA: *R* ^2^ = 0.24, *p* = 0.001; Supporting Information Figure A2B). In water they varied significantly with both season (PERMANOVA: *R* ^2^ = 0.34, *p* = 0.001; Supporting Information Figure A3C) and location (PERMANOVA: *R* ^2^ = 0.25, *p* = 0.001; Supporting Information Figure A3C), while tufa communities showed weaker but detectable seasonal (PERMANOVA: *R* ^2^ = 0.25, *p* = 0.001) and location differences (PERMANOVA: *R* ^2^ = 0.12, *p* = 0.052; Supporting Information Figure A3D).

On average, 46% of prokaryotic ASVs were shared between tufa and water, while 35% were unique to tufa and 18% to water (Supporting Information Figure A4A–D). In contrast, most microeukaryotic ASVs (80%) were unique to water, with only small fractions shared (9%) or unique to tufa (3%), except in autumn at Novakovića Brod, where 36% were unique to tufa (Supporting Information Figure A4E–H).

Similarity of prokaryotic communities decreased with geographic distance (3.5 km), showing comparable distance—decay in both water and tufa (Supporting Information Figure A5A,B). Microeukaryotic communities also declined in similarity, with a steeper decrease in tufa (Supporting Information Figure A5C,D).

Compositional stability of prokaryotic communities was slightly higher in water during summer and in tufa during autumn at Novakovića Brod, though significant temporal change was observed only in water communities at this site during autumn (*p* = 0.018; Supporting Information Figure A6). For microeukaryotes, stability was higher in water during summer and in tufa during autumn at Novakovića Brod, with significant change detected only in water communities at this site (*p* = 0.046; Supporting Information Figure A7).

Water prokaryotic communities were dominated by Actinobacteriota, Bacteroidota, and Proteobacteria in summer, with Cyanobacteria also abundant at Novakovića Brod, while Verrucomicrobiota dominated Prošćansko in autumn and Cyanobacteria at Novakovića Brod (Supporting Information Figure A8A). Tufa prokaryotic communities were consistently dominated by Proteobacteria, with Firmicutes also abundant, particularly at Prošćansko, and Bacteroidota increased abundances toward the end of the summer sampling (Supporting Information Figure A8B).

Water microeukaryotic communities were composed of Alveolata, Archaeplastida, Hacrobia, and Stramenopiles, with Opisthokonta prominent at Novakovića Brod in summer and Stramenopiles at Prošćansko in autumn (Supporting Information Figure A9A). Tufa microeukaryotic communities were dominated by Opisthokonta in summer at Prošćansko, while autumn samples showed higher abundances of Archaeplastida, Alveolata, and Stramenopiles at both sites (Supporting Information Figure A9B).

### 3.5 Colonized Tufa Microbial Communities

**Figure 4:**
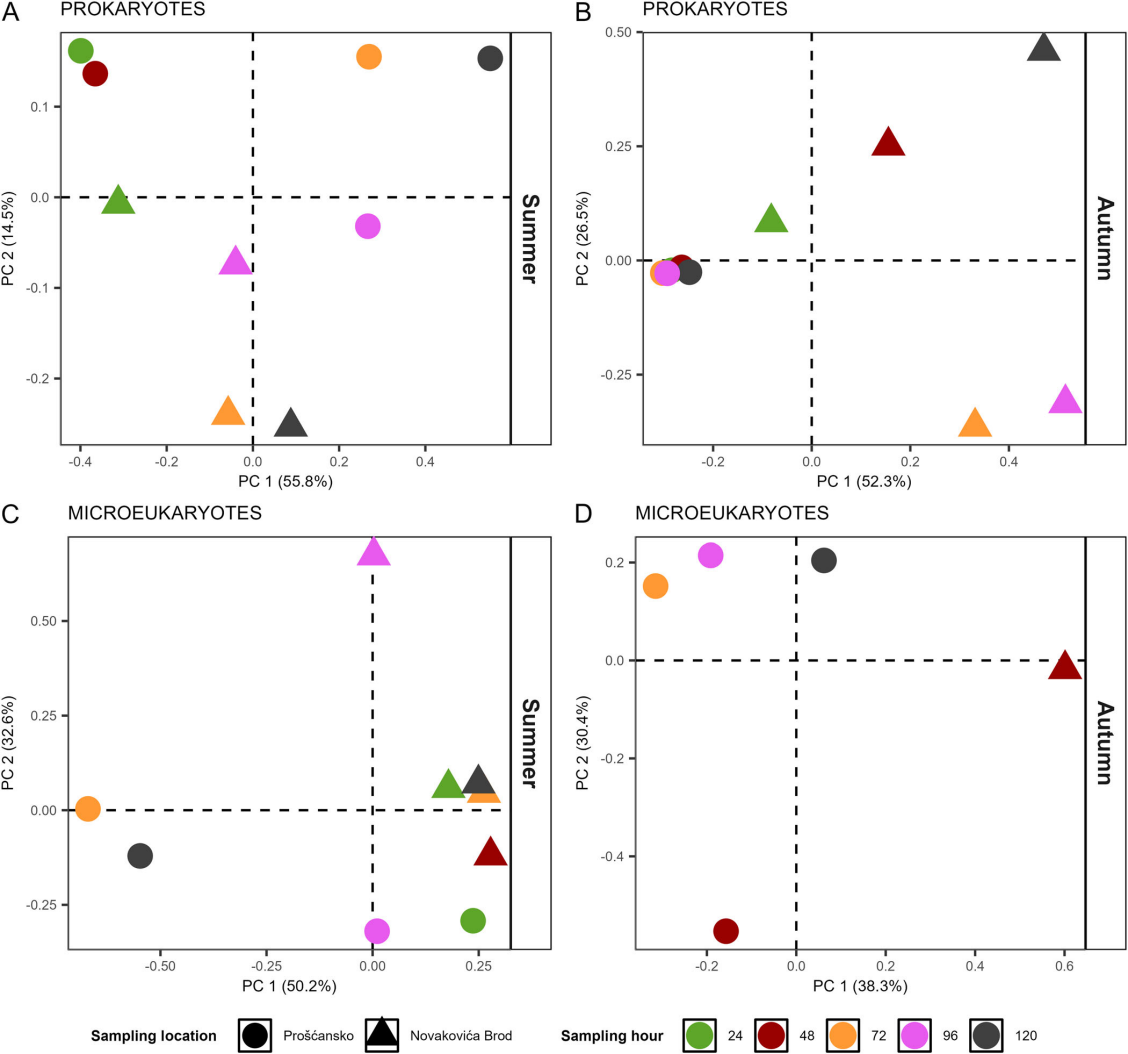
Significant difference in microbial communities of tufa deposits. Principal coordinate analysis ordination showing Bray–Curtis dissimilarity in prokaryotic (A, B) and microeukaryotic (C, D) community compositions throughout sampling hour across color coded by sampling hour and shape coded by sampling location (see legend). PC, polycarbonate.

Multivariate analysis showed no significant differences in tufa prokaryotic communities during summer (PERMANOVA: *R* ^2^ = 0.14, *p* = 0.292; Figure 4A), whereas in autumn they differed significantly between locations (PERMANOVA: *R* ^2^ = 0.41, *p* = 0.006; Figure 4B). In summer, Prošćansko samples grouped into early (24–48 h) and late clusters, while Novakovića Brod samples from the last two time points were most similar (Figure 4A). In autumn, Prošćansko samples formed a single cluster, whereas Novakovića Brod samples varied by sampling time (Figure 4B).

Tufa microeukaryotic communities differed significantly between locations in summer (PERMANOVA: *R* ^2^ = 0.33, *p* = 0.032; Figure 4C), but not in autumn (PERMANOVA: *R* ^2^ = 0.35, *p* = 0.2; Figure 4D). In summer, Novakovića Brod samples were similar except at 96 h, while Prošćansko samples separated into two clusters, 24 h with 96 and 72 h with 120 h (Figure 4C). In autumn, the Prošćansko sample at 48 h and the Novakovića Brod sample differed from the others (Figure 4D).

**Figure 5:**
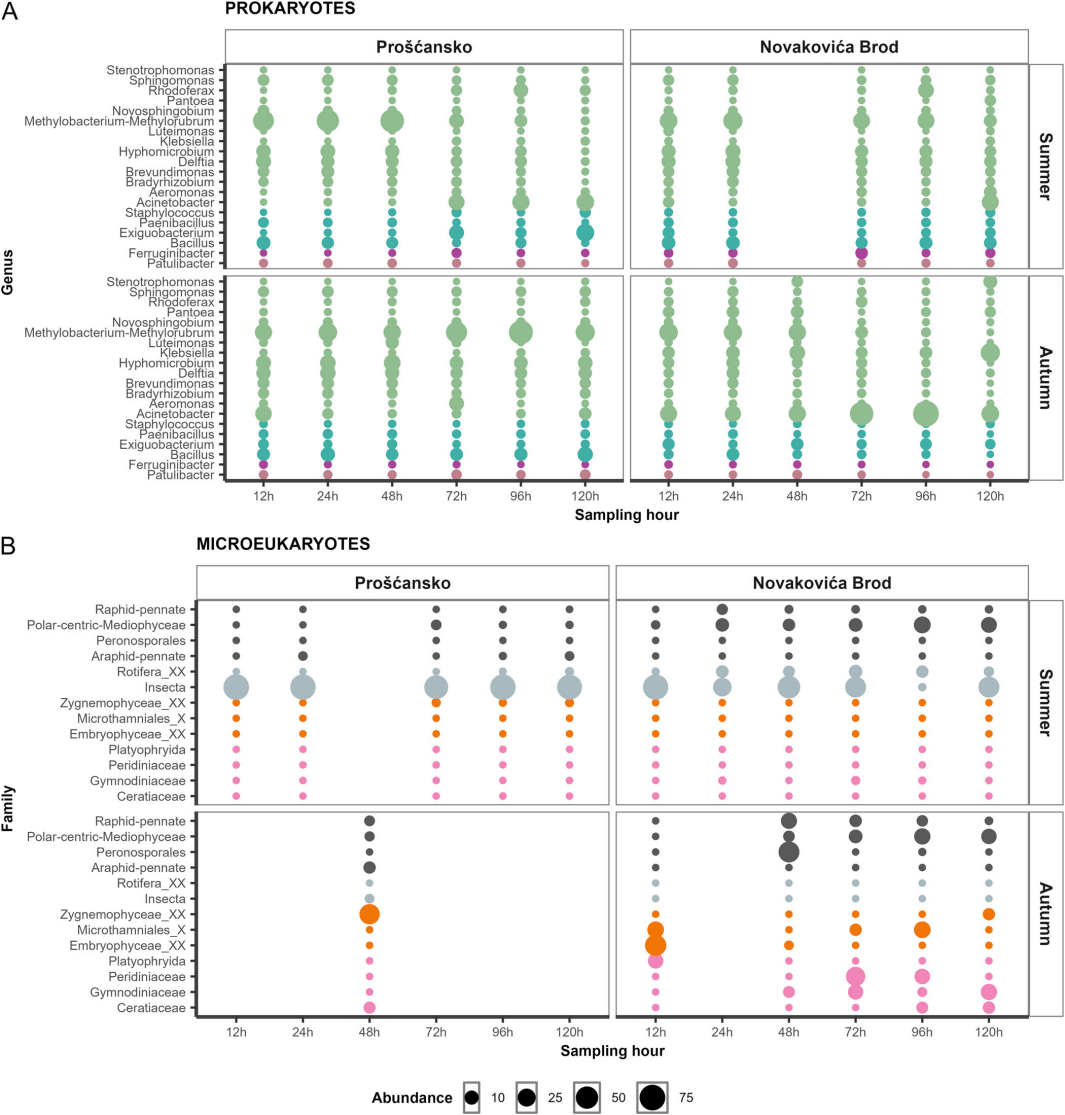
Microbial community composition in tufa biofilms. Relative abundance of the most abundant prokaryotic (A) and microeukaryotic (B) taxa in tufa biofilm samples divided by season and locations.

In summer, *Bacillus, Delftia, Hyphomicrobium*, and *Methylobacterium–Methylorubrum* dominated early tufa communities, but later their abundances declined, while those of *Exiguobacterium, Acinetobacter*, and *Rhodoferax* increased. In autumn, *Methylobacterium–Methylorubrum* remained dominant at Prošćansko, while *Acinetobacter* was most abundant at Novakovića Brod (Figure 5A).

In summer, Insecta absolutely dominated microeukaryotic tufa communities at Prošćansko, while at Novakovića Brod, Rotifera, Polar‐centric‐Mediophyceae, and Raphid‐pennate had higher abundances. In autumn, Zygnemophyceae dominated at Prošćansko, alongside Ceratiaceae, Araphid‐pennate, Polar‐centric‐Mediophyceae, and Raphid‐pennate, whereas community composition at Novakovića Brod shifted with sampling time (Figure 5B).

**Figure 6:**
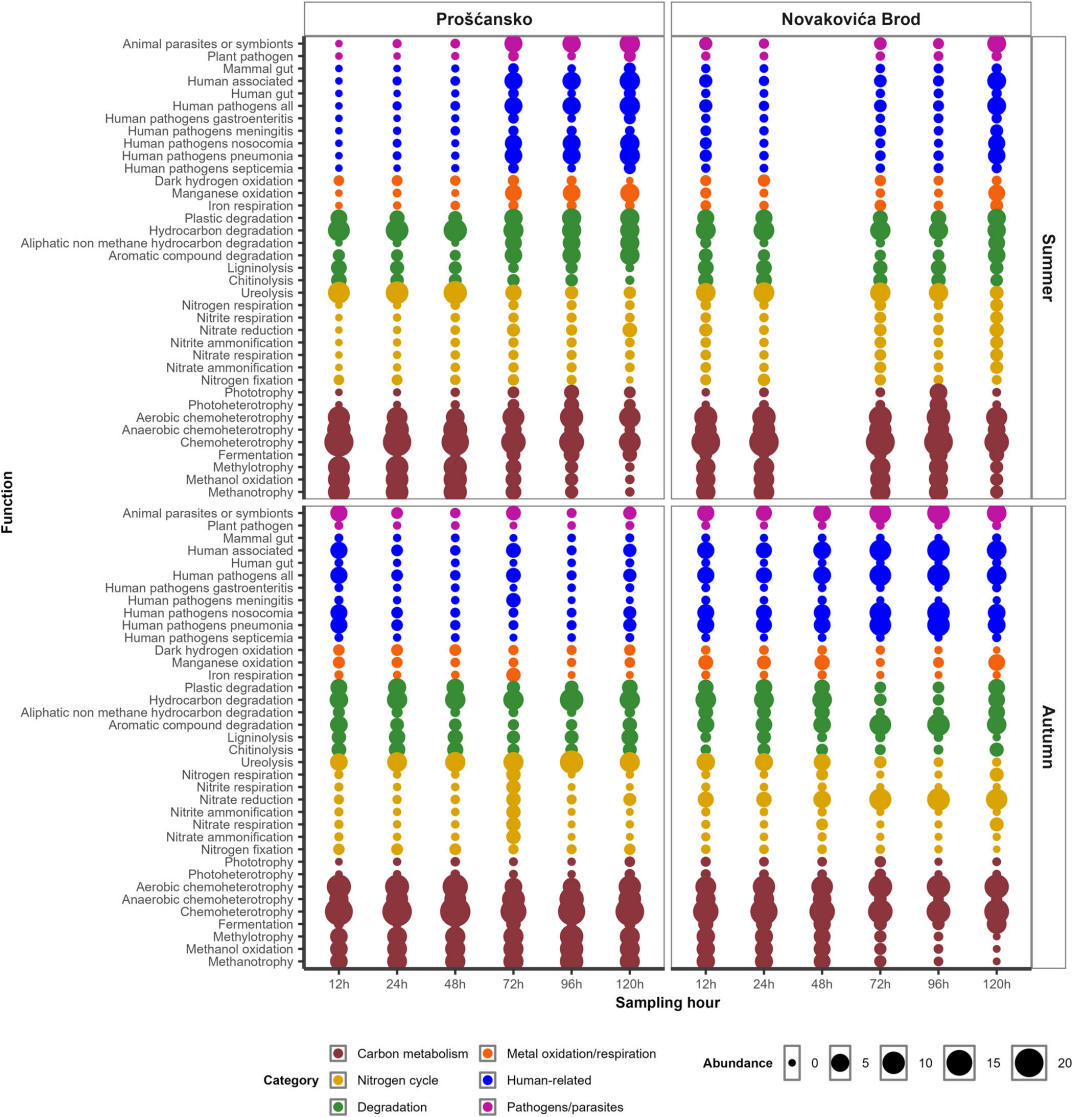
Functional potential of prokaryotic communities in tufa biofilms. Relative abundance of the functions examined by functional annotation analysis of prokaryotic communities in tufa biofilm samples, divided by season and locations.

Functional annotation analysis revealed that carbon metabolism functions dominated prokaryotic activity, with chemoheterotrophy prevalent across all seasons and locations. Methanotrophy, methanol oxidation, and methylotrophy were more common in summer and during the early sampling period (12–48 h) (Figure 6). Within the nitrogen cycle, ureolysis was consistently abundant, especially in early samples, while nitrate reduction increased in autumn at Novakovića Brod. Hydrocarbon degradation remained high in all samples, while aromatic compound degradation was more pronounced at Prošćansko in summer and at Novakovića Brod in autumn. Human‐related functions were more abundant at Novakovića Brod in autumn, while animal parasites or symbionts were more common at Prošćansko in summer and again at Novakovića Brod late in autumn sampling (Figure 6).

**Figure 7:**
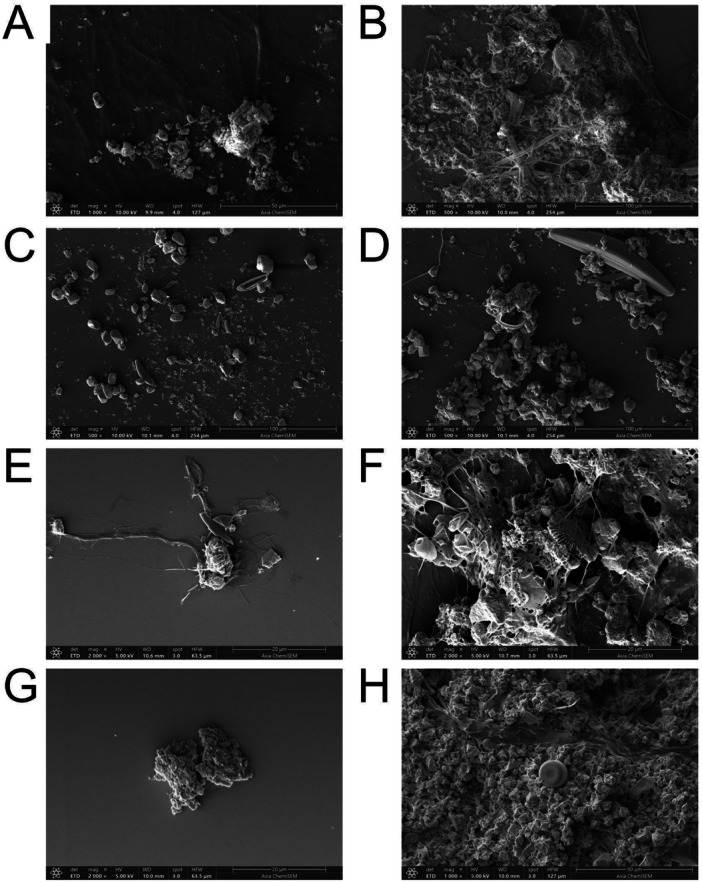
Scanning electron microscopy (SEM) analysis of temporal and spatial development of tufa biofilms. SEM images of tufa biofilm samples precipitated on glass slides: summer sampling at Prošćansko Lake after 12 h (A) and 120 h (B) and at Novakovića Brod after 12 h (C) and 120 h (D); autumn sampling at Prošćansko Lake after 12 h (E) and 120 h (F) and at Novakovića Brod after 12 h (G) and 120 h (H).

Initial tufa biofilms (12 h) were structurally simple, with few diatoms, limited EPS, and minimal CaCO_3_ entrapment (Figure 7). By 120 h, biofilms became more complex, with denser EPS, higher diatom abundance, and visible CaCO_3_ crystal accumulation (Figure 7). SEM/EDS mapping confirmed this progression, showing silicon dominance at 12 h and increased calcium and oxygen at 120 h (Supporting Information Figure A10).

## 4 Discussion

Tufa formation relies on microbial activity, where EPS produced by periphyton traps CaCO_3_ particles. After 12 h, samples showed simple biofilms with diatoms, limited EPS, and minimal CaCO_3_ capture (Figure 7), accompanied by high silicon signals from diatom frustules (Supporting Information Figure A10). By 120 h, EPS density, diatom abundance, and CaCO_3_ accumulation increased (Figure 7), paralleled by higher calcium and oxygen signals, indicating active mineralization (Supporting Information Figure A10). These findings highlight microbial and EPS roles in the gradual carbonate buildup during early tufa formation.

### 4.1 Differences in Tufa and Water Microbial Communities

Tufa samples showed prokaryotic richness and diversity comparable to, and in some cases higher than water samples (Figure 2). Water communities varied by season and location, with higher values at Prošćansko in summer and at Novakovića Brod in autumn, a pattern also noted previously (Čačković et al. 2023). In contrast, microeukaryotic diversity was lower in tufa than in water, consistent with earlier studies (Parfenova et al. 2013).

Beta diversity revealed clear differences between sample types already after 12 h (Figure 3A). Tufa prokaryotic communities remain stable across locations and seasons, dominated by Proteobacteria and Firmicutes (Figure 3B), early colonizers in carbonate‐rich habitats (Schneider et al. 2015; Arp et al. 2010). In contrast, water communities were more variable between sites and seasons, reflecting environmental influences and a broader mix of bacterial phyla (Čačković et al. 2023). Interestingly, Cyanobacteria, typically described as dominant members of the tufa deposit community (Schneider et al. 2015), were found in low abundance, with slightly higher abundance observed in the water samples (Figure 3B). Initial microeukaryotic communities were dominated by Alveolata, Archaeplastida, and summer‐abundant Opisthokonta (Figure 3D), which includes several protist lineages, fungi, and animals, including Insceta (Bonacolta et al. 2024). Later samples showed stronger spatial structuring in both prokaryotic and microeukaryotic communities, with seasonal differences evident in water samples but not in tufa, likely due to minimal changes in environmental conditions (Gautam et al. 2022; Supporting Information Figure A1), with geographic distance (Supporting Information Figure A5) influencing community composition in both water and tufa (Čačković et al. 2023).

The continuous contact between water and tufa facilitated microbial exchange, as evidenced by the large number of shared ASVs (Supporting Information Figure A4), particularly in the early stages when the biofilm‐forming community is not yet specialized and includes microbe settling from the plankton, surface attachment, and their interactions during biofilm development (Peng et al. 2018). Over time, tufa communities rapidly stabilized, forming specialized biofilms, while water communities remained more dynamic and seasonally driven (Supporting Information Figures A6 and A7).

### 4.2 Differences in Colonized Tufa Microbial Communities

Succession within colonized tufa biofilms reflected functional roles essential for tufa barrier formation and stabilization. Early stages were dominated by heterotrophic Proteobacteria, Firmicutes, and Bacteroidota (Supporting Information Figure A8), with *Bacillus, Delftia, Hyphomicrobium,* and *Methylobacterium* (Figure 5A) promoting mineral nucleation on biofilm matrices, creating structure of early tufa layers (Castanier et al. 1999; Dupraz et al. 2004; Beraldi‐Campesi et al. 2012). As biofilms matured, communities shifted toward *Acinetobacter* and *Rhodoferax* (Figure 5A), consistent with increasing metabolic complexity and organic matter degradation (Beraldi‐Campesi et al. 2012). Site‐specific patterns, including higher *Acinetobacter* in autumn at Prošćansko (Figure 5A), suggest dynamic or disturbed tufa growth conditions linked to higher terrestrial nutrient loads (Nemec et al. 2021).

Microeukaryotic succession was equally dynamic. In summer, Insecta dominated at Prošćansko, while benthic diatoms and rotifers were more prominent at Novakovića Brod (Figure 5B), indicating their role in early biofilm development and stabilization (Bondoc‐Naumovitz and Cohn 2021; Cardozo‐Mino et al. 2023; Jiang et al. 2023). In autumn, diatoms (Ceratiaceae, Araphid‐pennate, Polar‐centric Mediophyceae and Raphid‐pennate) with Zygnemophyceae (Figure 5B) became dominant, emphasizing their contribution to EPS production (Sivakumar et al. 2025) and CaCO_3_ entrapment (Souza‐Egipsy et al. 2006).

During summer, tufa prokaryotic communities differed between early and late sampling points (Figure 4A). Early samples were enriched with *Bacillus* and *Delftia* (Figure 5A), associated with chemoheterotrophy, ureolysis, and hydrocarbon degradation (Zhang et al. 2022; Figure 6), with *Hyphomicrobium* and *Methylobacterium–Methylorubrum* (Figure 5A), associated with methanotrophy, methanol oxidation, and methylotrophy methanol oxidation (Alessa et al. 2021; Figure 6). In late samples, *Exiguobacterium* and *Rhodoferax* dominated (Figure 5A), contributing to the consistently high hydrocarbon degradation (Wang et al. 2023; Figure 6).

During autumn, tufa communities differed significantly by location (Figure 4B). At Prošćansko, functions such as chemoheterotrophy, methanotrophy, and methylotrophy (Figure 6) were more abundant, in line with higher *Methylobacterium–Methylorubrum* (Seppey et al. 2023; Figure 5A). At Novakovića Brod, functions for aromatic compound degradation, nitrate reduction, and human gut with animal parasites or symbionts were dominant (Figure 6), with higher abundance of *Acinetobacter* (Figure 5A), a genus linked to human‐ and animal‐associated functions (Neogi et al. 2014).

## 5 Conclusion

This study highlights distinct patterns in the composition, diversity, and functions of microbial communities associated with tufa and surrounding water across spatial and temporal scales in karst streams. Prokaryotic communities in tufa exhibited early stability and structural specificity, shaped by initial colonizers, such as *Bacillus*, *Delftia*, *Hyphomicrobium*, and *Methylobacterium–Methylorubrum*, which play essential roles in initiating carbonate precipitation processes. As biofilms matured, a shift toward genera capable of complex organic matter degradation, such as *Acinetobacter* and *Rhodoferax*, indicated the development of functionally specialized communities necessary for long‐term tufa deposition.

Microeukaryotic communities displayed more pronounced spatial and seasonal variability, with taxa such as diatoms (Raphid‐pennate, Mediophyceae, Ceratiaceae) and Zygnemophyceae contributing to EPS production critical for carbonate entrapment. Differences between sites, particularly between Prošćansko and Novakovića Brod, reflect the influence of local environmental conditions, including vegetation input and hydrological dynamics, on microbial structure and function.

These results provide insight into understanding of the microbial dynamics involved in tufa barrier formation, illustrating how distinct prokaryotic and microeukaryotic communities adapt to environmental conditions and collectively drive the biogeochemical processes essential for calcium carbonate deposition in freshwater systems.

## Author Contributions

**Andrea Čačković:** conceptualization, formal analysis, investigation, methodology, visualization, writing – original draft. **Andrijana Brozinčević:** conceptualization, data curation, methodology, writing – review and editing. **Marija Mirosavljević:** formal analysis, investigation. **Sandi Orlić:** conceptualization, data curation, funding acquisition, project administration, resources, writing – review and editing.

## Ethics Statement

The authors have nothing to report.

## Conflicts of Interest

None declared.

## Supporting information

Appendices.

## Data Availability

Raw sequence reads were deposited in the NCBI repository under BioProject accession number PRJNA1359380.
