# Supplementary material for: Microbial Community Dynamics in Early Tufa Biofilms
Source: Microbiologyopen. 2025 Nov 17;14(6):e70153. doi: 10.1002/mbo3.70153 (PMC12620666; doi:10.1002/mbo3.70153)
Supplement: Supplementary file 1 — Appendices. [file MBO3-14-e70153-s001.pdf]

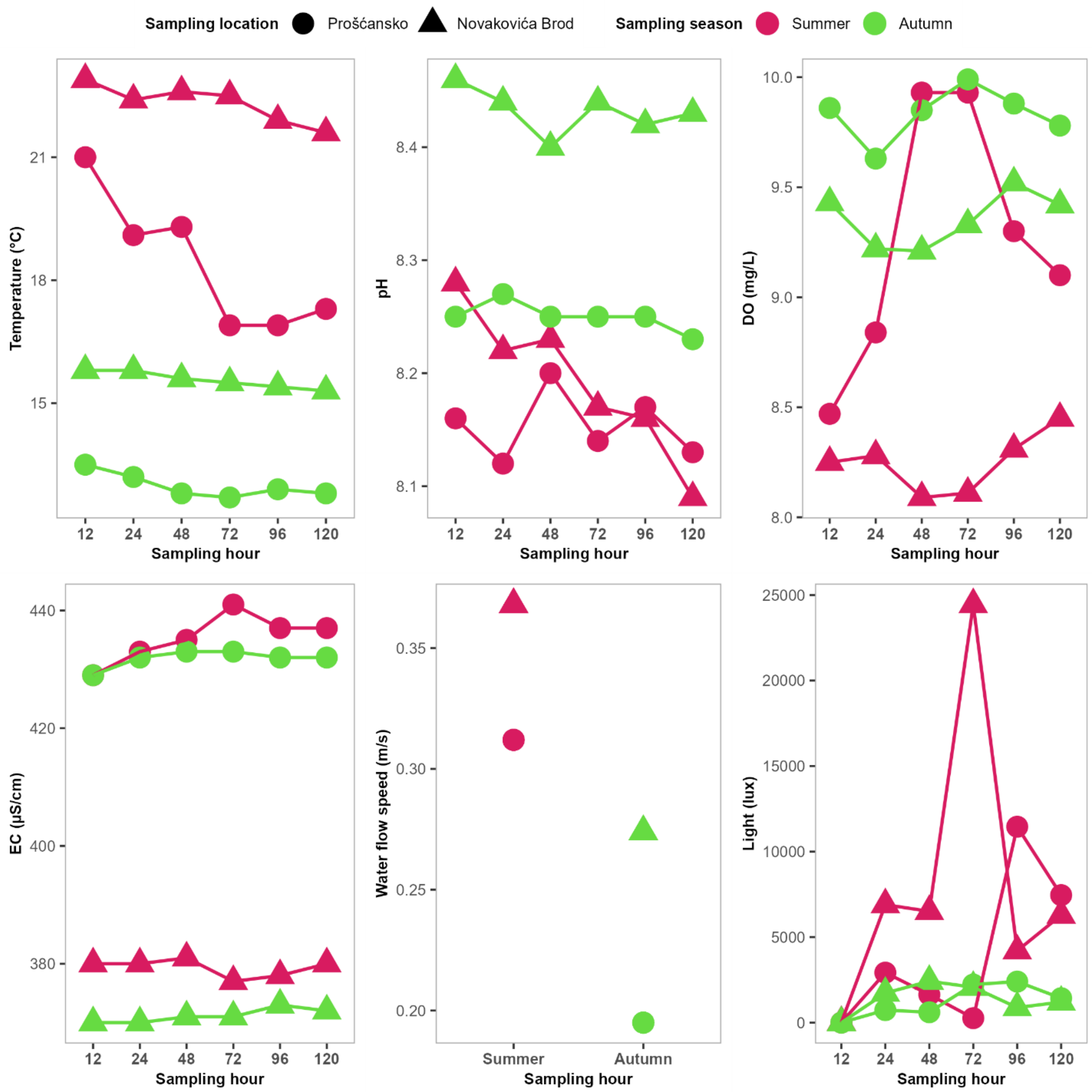

Figure A1. Environmental parameters of water measured at the sampling locations during two sampling seasons.

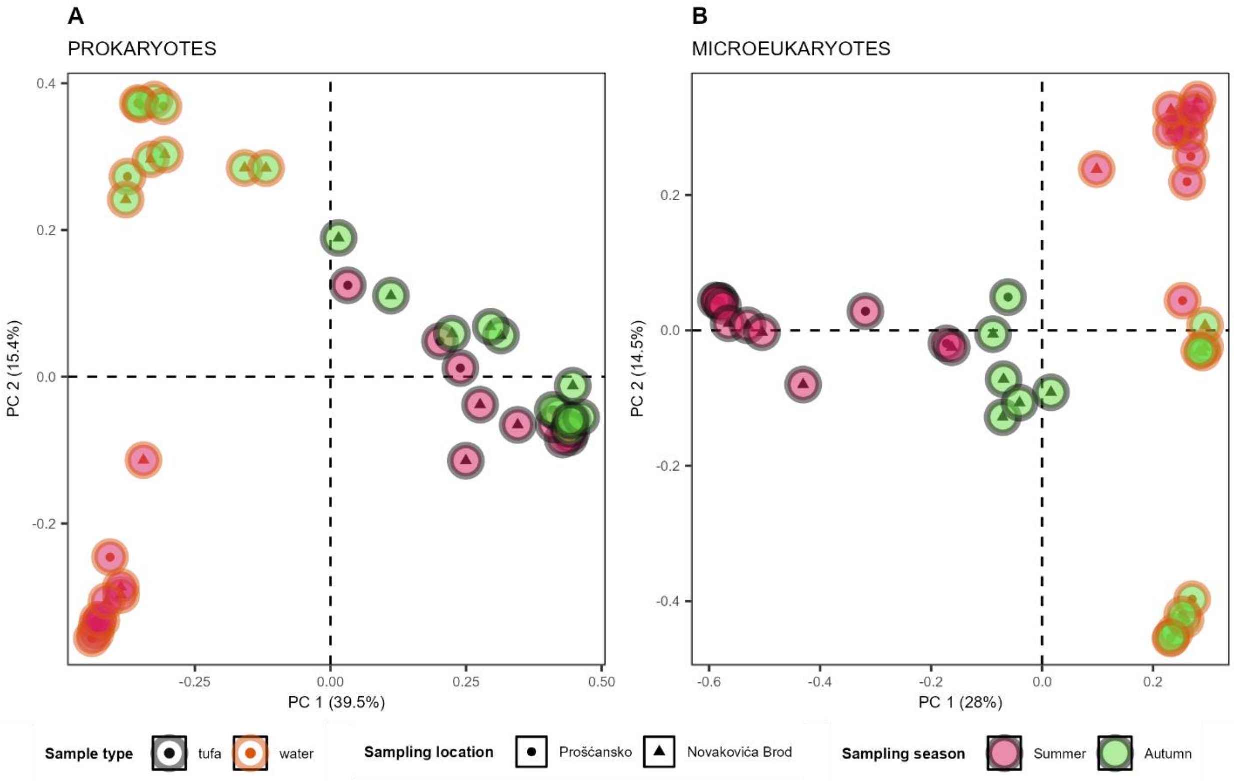

Figure A2. Principal coordinate analysis (PCoA) ordination showing Bray-Curtis dissimilarity in prokaryotic (A) and microeukaryotic (B) community compositions across different sample types, color coded by sampling season and shape coded by sampling location (see legend).

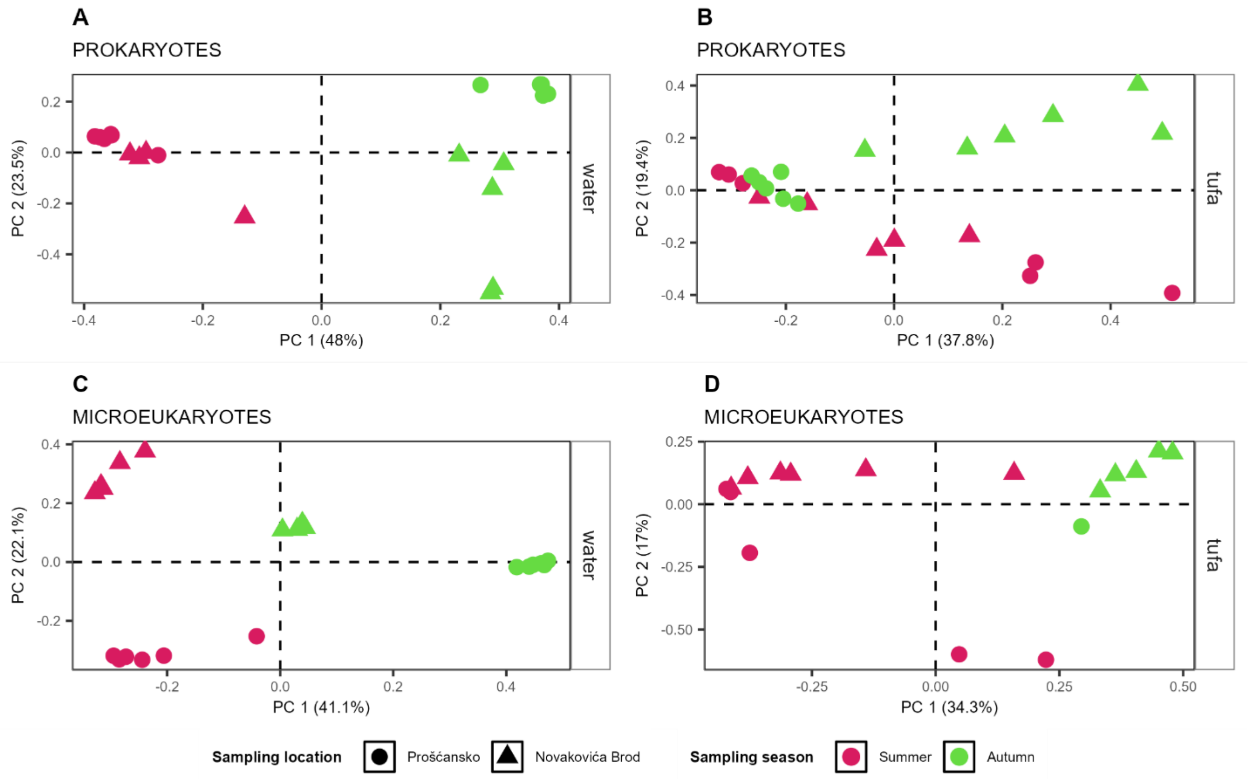

Figure A3. Principal coordinate analysis (PCoA) ordination showing Bray-Curtis dissimilarity in prokaryotic (A-B) and microeukaryotic (C-D) community compositions across water and tufa samples, color coded by sampling season and shape coded by sampling location (see legend).

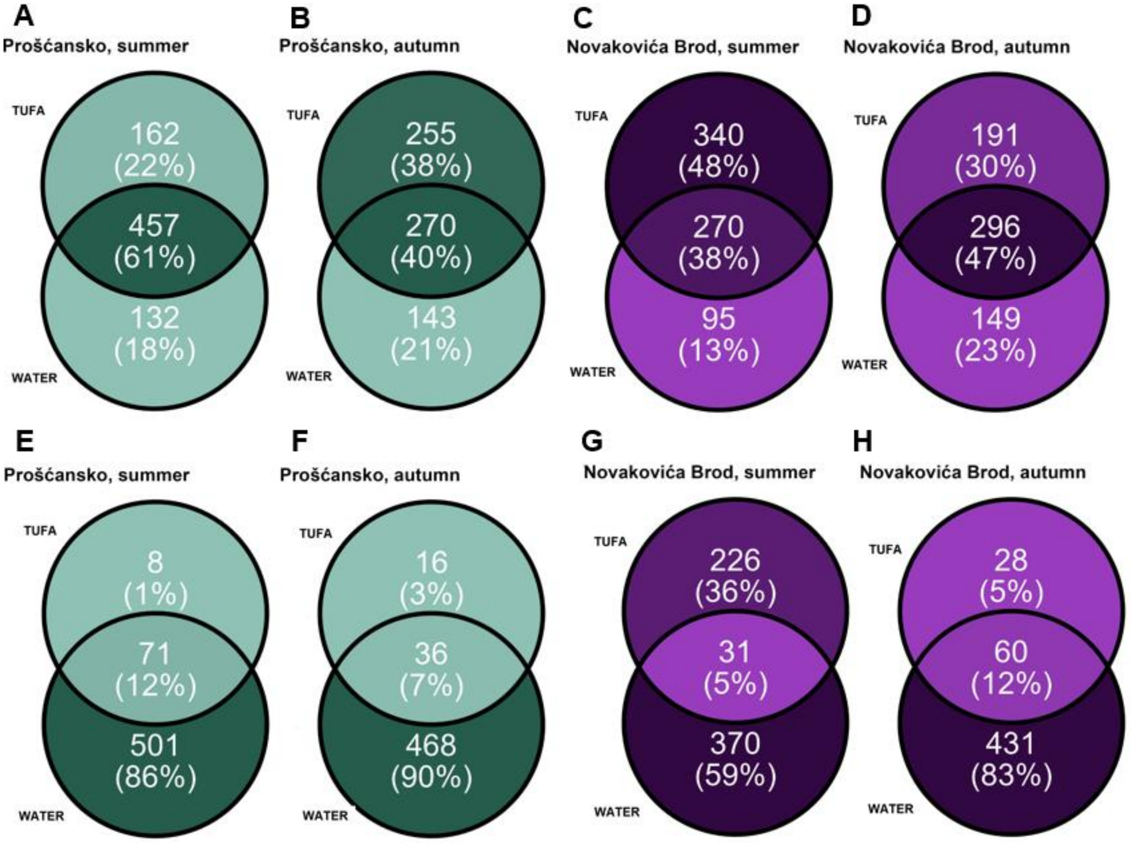

Figure A4. Venn diagram representing the number of shared and unique prokaryotic ASVs between tufa and water at Prošćansko location during summer (A) and autumn (B) and at Novakovića Brod location during summer (C) and autumn (D), as well microeukaryotic ASVs between tufa and water at Prošćansko location during summer (E) and autumn (F) and at Novakovića Brod location during summer (G) and autumn (H).

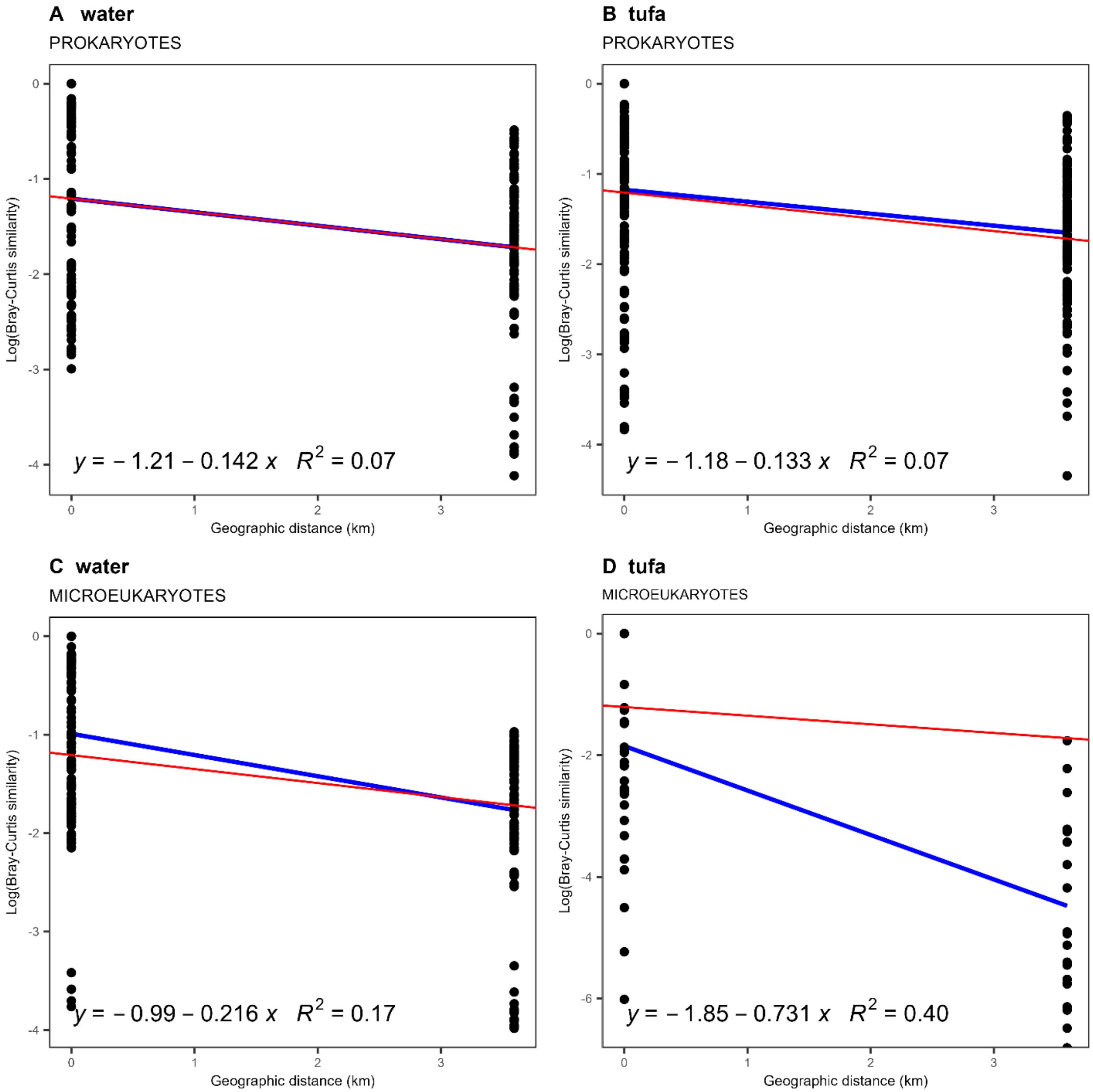

Figure A5. Pairwise Bray-Curtis community similarity between samples with respect to geographic distance (km). (A) Prokaryotic communities in water samples. (B) Prokaryotic communities in tufa samples. (C) Microeukaryotic communities in water samples. (D) Microeukaryotic communities in tufa samples. Blue lines illustrate linear models computed for the subset of samples considered, and red lines represent the overall linear regression when including all the samples.

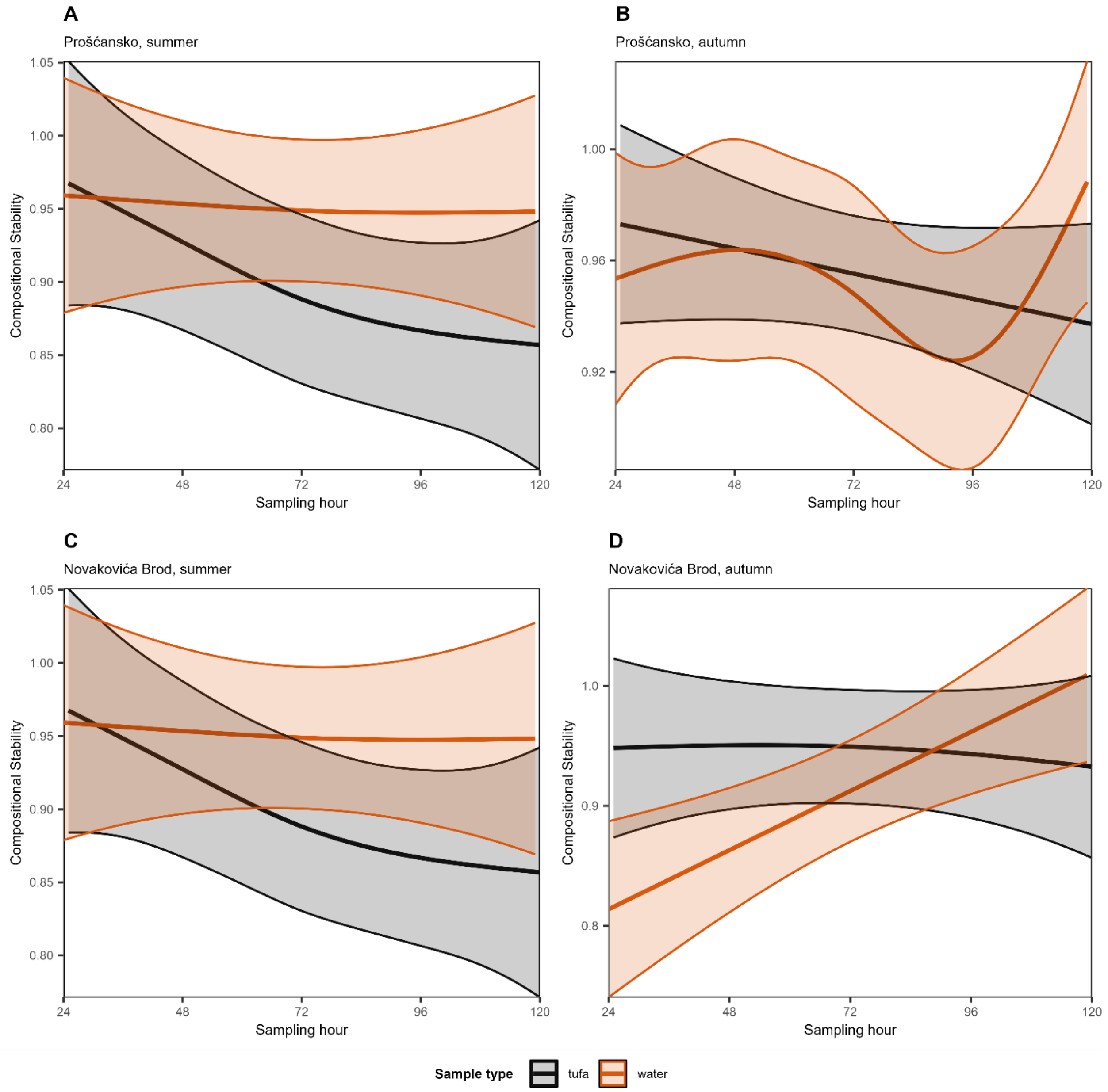

Figure A6. Dynamics of the prokaryotic community stability in water and tufa samples throughout sampling period at Prošćansko location during summer (A) and autumn (B) and at Novakovića Brod location during summer (C) and autumn (D).

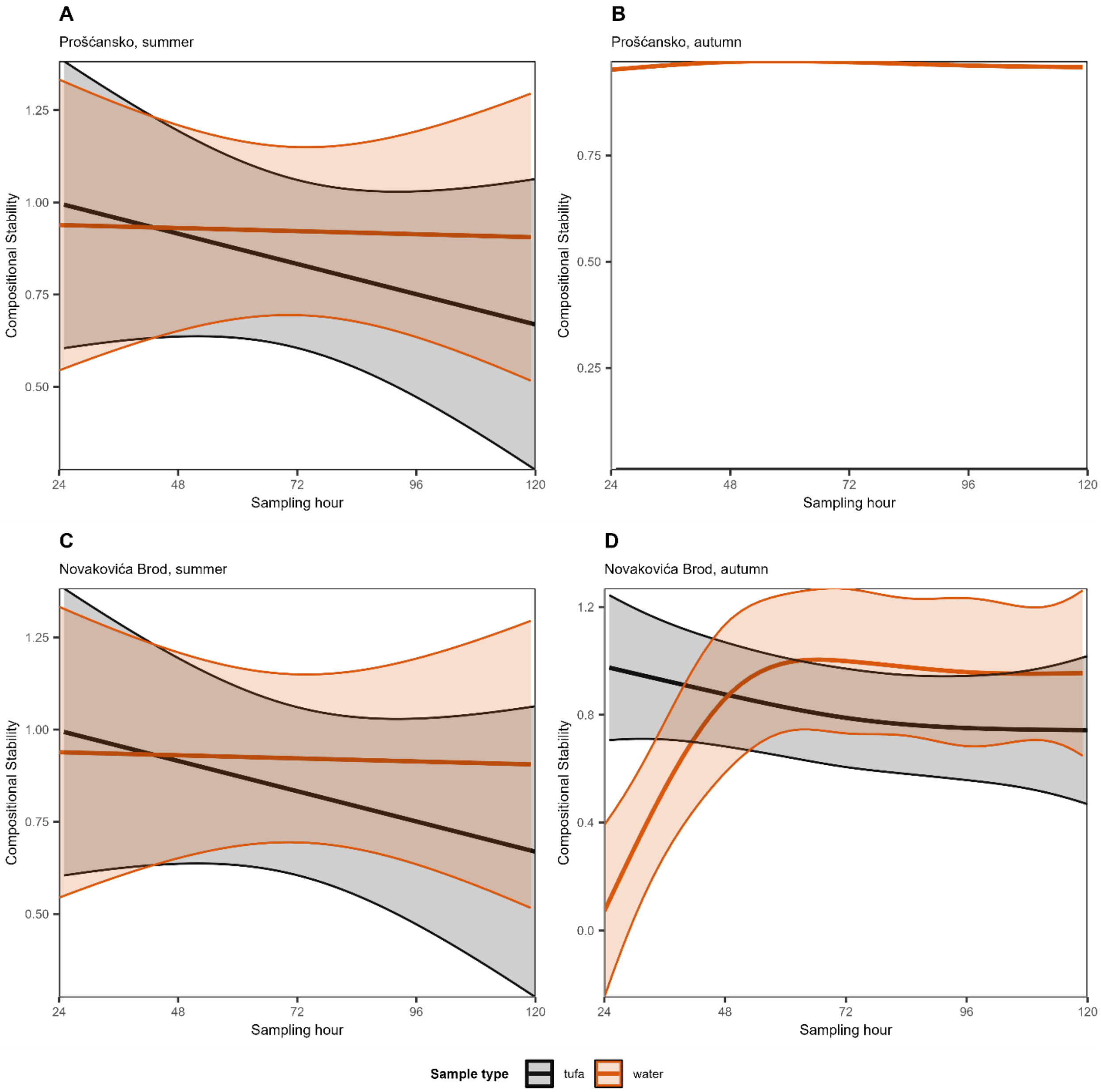

Figure A7. Dynamics of the microeukaryotic community stability in water and tufa samples throughout sampling period at Prošćansko location during summer (A) and autumn (B) and at Novakovića Brod location during summer (C) and autumn (D).

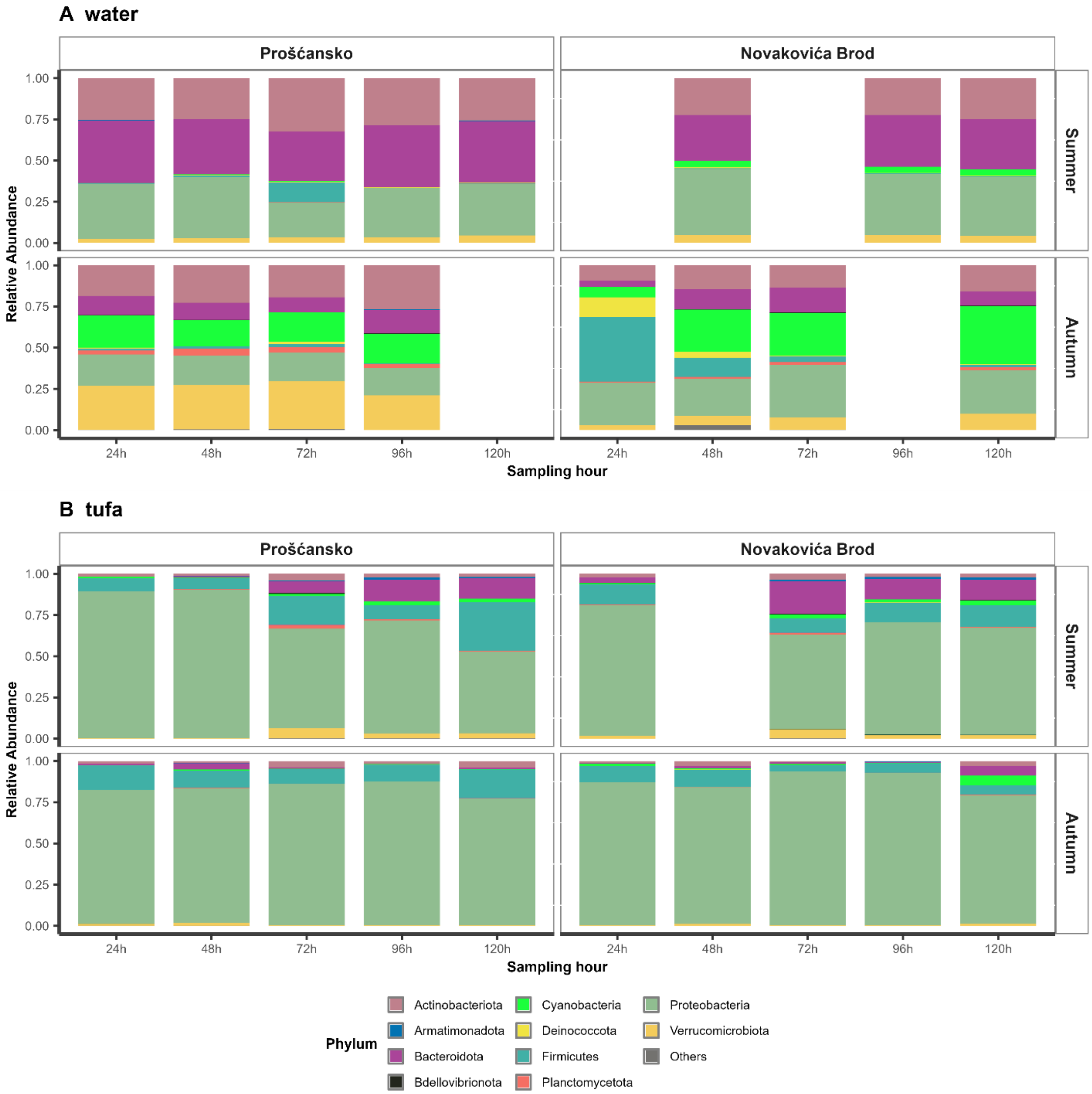

Figure A8. Average relative abundance of the prokaryotic community structure (phylum level) in water (A) and tufa (B) at sampling locations during two sampling seasons. Taxa are colored by phylum. The “other’’ group contains phyla with a relative abundance of less than 1%.

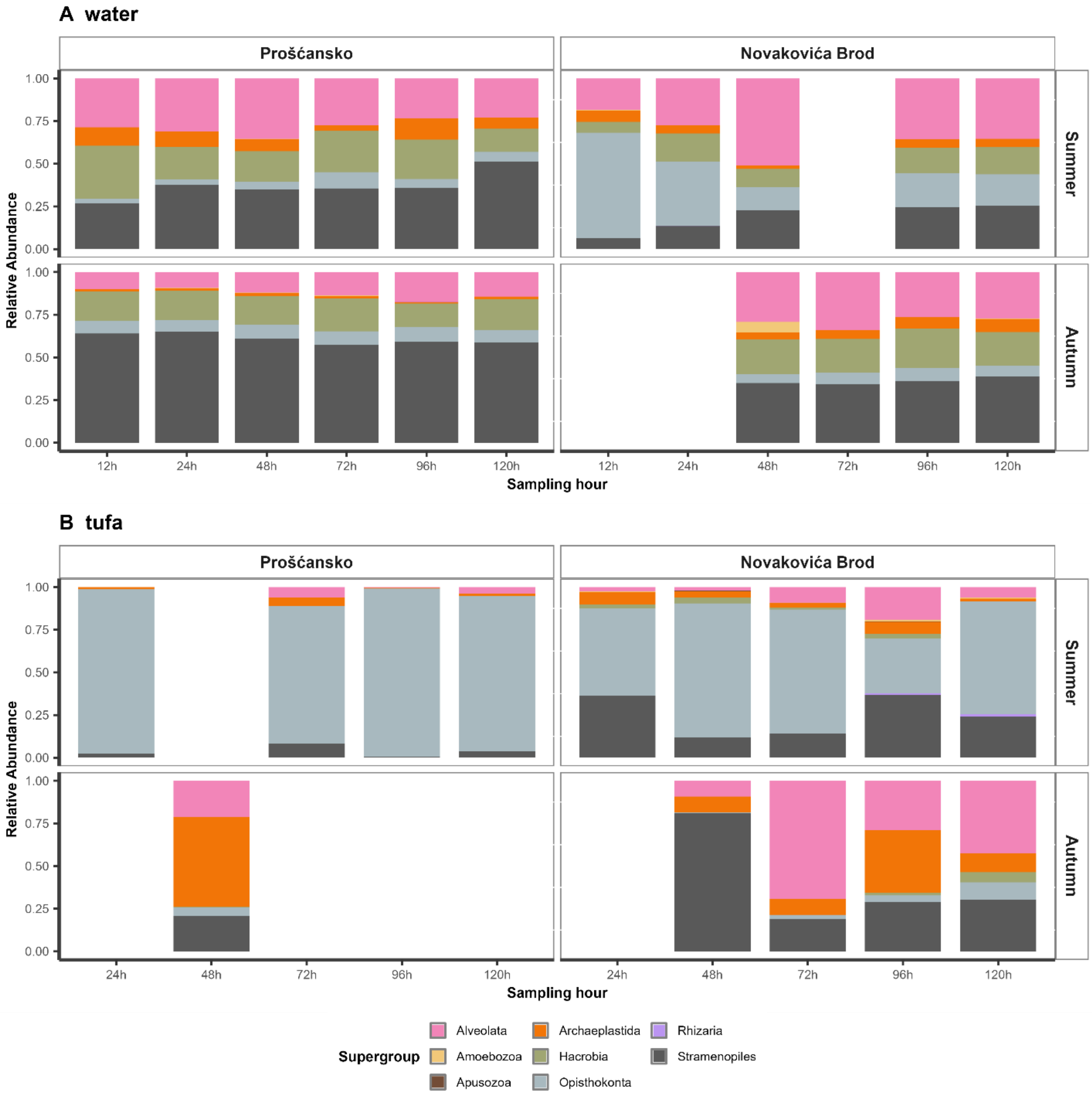

Figure A9. Average relative abundance of the microeukaryotic community structure (supergroup level) in water (A) and tufa (B) at sampling locations during two sampling seasons. Taxa are colored by supergroup. The "other'' group contains phyla with a relative abundance of less than 1%.

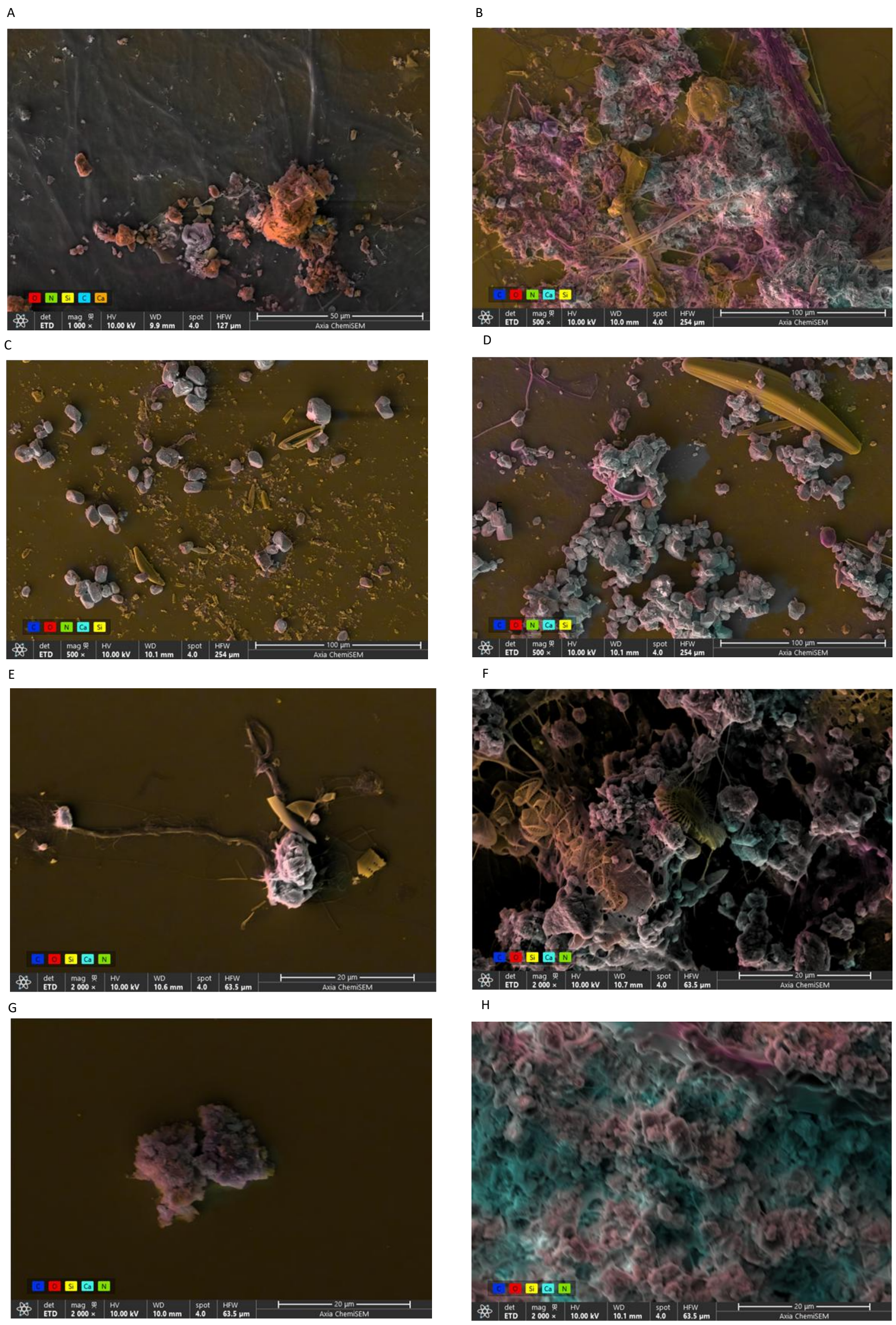

Figure A10. SEM-EDS images of tufa biofilm samples precipitated on glass slides: Summer sampling at Prošćansko Lake after 12 hours (A) and 120 hours (B), and at Novakovića Brod after 12 hours (C) and 120 hours (D); Autumn sampling at Prošćansko Lake after 12 hours (E) and 120 hours (F), and at Novakovića Brod after 12 hours (G) and 120 hours (H).
